# Executive Functions in Alzheimer Disease: A Systematic Review

**DOI:** 10.3389/fnagi.2018.00437

**Published:** 2019-01-15

**Authors:** Angela Guarino, Francesca Favieri, Ilaria Boncompagni, Francesca Agostini, Micaela Cantone, Maria Casagrande

**Affiliations:** ^1^Dipartimento di Psicologia, Università di Roma “Sapienza”, Rome, Italy; ^2^Dipartimento di Psicologia Dinamica e Clinica, Università di Roma “Sapienza”, Rome, Italy

**Keywords:** alzheimer's disease, executive functions, wisconsin card sorting test, stroop task, go/no-go task, flanker task

## Abstract

Alzheimer's disease is a severe irreversible syndrome, characterized by a slow and progressive cognitive decline that interferes with the standard instrumental and essential functions of daily life. Promptly identifying the impairment of particular cognitive functions could be a fundamental condition to limit, through preventive or therapeutic interventions, the functional damages found in this degenerative dementia. This study aims to analyse, through a systematic review of the studies, the sensitivity of four experimental paradigms (Wisconsin Card Sorting Test, Stroop Task, Go/No-Go Task, and Flanker Task) considered as golden standard instruments for executive functions assessment in elderly subjects affected by Alzheimer dementia. This review was carried out according to the PRISMA method. Forty-five studies comparing the executive performance of patients with Alzheimer's dementia (diagnosed according to different classification criteria for dementia) and healthy elderly patients both over the age of sixty, were selected. For the research, PubMed, PsycINFO, PsycArticles databases were used. The study highlighted the importance of using standard protocols to evaluate executive dysfunction in Alzheimer's disease. The Stroop task allows discriminating better between healthy and pathological aging.

## Introduction

The World Health Organization defines dementia as a “loss of intellectual capability of such severity as to interfere with the social or occupational functioning” (World Health Organization, 2010). It is a complex irreversible and chronic syndrome (Knapp et al., 2007) in which a slow and progressive cognitive decline affects the typical performance of the practical and essential functions of daily life (Boccardi, 2007).

The typical symptoms of dementia involve different cognitive domains, such as memory, spatial and temporal orienting, language and learning, comprehension, and communication skills (World Health Organization, 1992); moreover, alterations in emotional control, motivation and social behavior are often present (Kipps et al., 2009). This problem appears to be increasing in the general population (World Health Organization, 2012). It is estimated that about 46.8 million people worldwide, in 2015 had dementia; continually growing numbers that are expected to double every 20 years, reaching estimates of 74.7 million people in 2030 (Prince et al., 2016).

The calculation of the annual health costs related to dementias are similar to those of heart diseases, and they are much higher than those referred to cancer. These data would place dementia among the most expensive diseases in society (Prince et al., 2016). Most of these costs are attributable to home care and long-term institutionalization.

The most widespread form of dementia is Alzheimer's Dementia (AD), which is one of the main risk factors for death in the affected individuals (Alzheimer's Association, 2016). The average duration of the disease varies between 4 and 8 years, although some patients may survive up to 20 years after the onset of the AD (Xie et al., 2008). Alzheimer dementia is estimated to have increased by 35.4% in 2015, significantly raising the specific costs of this disease (Prince et al., 2016).

Alzheimer's disease includes a pre-dementia and a dementia phase (Taylor and Thomas, 2013). Pre-dementia represents the initial period of the disorder, in which the first symptoms associated with episodic memory loss begin (starting with the removal of the most recent memories and experiences), symptoms that however do not interfere with the management of the activities of the daily life (Förstl and Kurz, 1999). However, in this Mild Cognitive Impairment condition (MCI), not amnesic dysfunctions are also reported (Hodges et al., 2006). With the progression of the disease and the transition to the actual dementia phase, in addition to a worsening of the memory symptoms (which begin to affect even the most ancient memories and experiences), linguistic and spatial orienting deficits emerge that involves a severe functional difficulty (Hodges et al., 2006). High levels of anxiety and a general lack of motivation complete the clinical profile of AD (Steinberg et al., 2008; Dening and Sandilyan, 2015). On the other hand, in this first phase the procedural memory is still relatively preserved, but with the aggravation of the disease, there is a complete compromise of the entire memory domain (Pucci, 2004).

Balota and Faust (2002) have reported that individuals with AD present specific difficulties in selecting relevant information by separating them from irrelevant ones, highlighting their difficulty in dividing attention among multiple stimuli and in the attentional control. Likewise, lexical and semantic abilities would seem to be compromised, while phonological and syntax abilities would seem to be relatively conserved. As the disease progresses, the vocabulary tends to become impoverished and phonemic, and semantic paraphasias begin to appear, with a diminishment of expressive and understanding abilities (Pucci, 2004). In addition to language, the skills of spatial and temporal orienting, cognitive control of behavior, visuomotor integration, and executive functions are strongly compromised (Pucci, 2004).

Alzheimer's disease is characterized by a complex neuropsychological profile, associated with the gradual degeneration of the various cortical areas affected by this pathology. In the AD, the entorhinal cortex and the hippocampus seem to be compromised initially, that is, the structures involved in recording and consolidating information and in episodic memory (Du et al., 2001). Moreover, some studies have shown that patients with Alzheimer's disease showed severe lesions in the hippocampal and parahippocampal regions and the medial temporal lobe (Prvulovic et al., 2002; Machulda et al., 2003).

The AD, except the rare forms caused by genetic anomalies, derives from the presence and interaction of different conditions (Ngandu et al., 2015). Late age (Hebert et al., 2013), familiarity (Green et al., 2002) and the inheritance of the APOE-4 gene (Farrer et al., 1997) represent the risk factors most associated with the AD. Smoking, obesity (Beydoun et al., 2014), diabetes (Reitz et al., 2011), low levels of education and the inability to remain socially and mentally active, making it impossible to rely on their reserves cognitive (Wang et al., 2012) that, when are low, would be included among the risk factors indirectly associated with Alzheimer's disease. Regular physical activity (Sofi et al., 2011), a diet low in saturated fats (Loef and Walach, 2012), good cardiovascular health and the absence of brain lesions (McKee et al., 2013) represent protective factors for cognitive decline.

## Executive Functions in Alzheimer's Disease

Executive functions represent a wide range of active cognitive processes, which allow responding in the appropriate way to environmental stimuli. This “umbrella term” includes verbal reasoning, problem-solving, planning, the ability to maintain sustained attention, resistance to interference, multitasking, cognitive flexibility, and the ability to cope with novelty (Stuss and Benson, 1986; Shallice, 1988; Damasio, 1995; Stuss et al., 1995; Grafman and Litvan, 1999; Burgess et al., 2000). To facilitate research in the field of Executive Functions, several authors (Miyake et al., 2000; Lehto et al., 2003; Diamond, 2013) have developed a tripartite classification that consists of:

- Inhibition, including inhibitory control, self-control (behavioral inhibition), and interference control (selective attention and cognitive inhibition). It includes the voluntary inhibition of dominant or automatic responses (Miyake et al., 2000) and would allow controlling behavior, thoughts and emotions, as well as attentional aspects, with the aim to respond appropriately to the needs of the task and specific objectives (Diamond, 2013);
- Updating, which allows keeping in mind and manipulating information. It involves the updating and the monitoring of the representations collected in the working memory (involvement of the Dorsolateral Prefrontal Cortex; Miyake et al., 2000), which allow responding appropriately to external tasks or stimuli, thanks to the processing of relevant information (Miyake et al., 2000);
- Cognitive flexibility (set-shifting), which allows modifying one's behavioral response to external stimuli (Baddeley and Hitch, 1994; Smith and Jonides, 1999; Diamond, 2013). It is characterized by the attentional shift between tasks or between different mental operations. This mechanism is commonly regarded as disengagement from an irrelevant task with subsequent anchorage on a relevant task to pursue a particular objective (Miyake et al., 2000). Diamond (2013) referring to this specific executive function uses the term Cognitive Flexibility, which allows underlining the ability to change the individual perspective not only from a spatial point of view but also by interpersonal and thoughtful perspectives.

Until the last 20 years, deficits in executive functions were rarely considered in the early stages of Alzheimer's disease (Allain et al., 2013). Some studies suggested that these were relatively preserved during the pre-clinical phase of the disorder (Broks et al., 1996; Razani et al., 2001). However, over the last years, this view has changed, and more recent studies have confirmed the presence in the AD of early impairment in a variety of tasks aimed at investigating executive functions (Binetti et al., 1996; Amieva et al., 2002; Bondi et al., 2002). These findings confirm that in the Alzheimer's disease executive functions are impaired from the early stages (Levy et al., 2002), primarily due to degeneration of the prefrontal cortex (Salat et al., 2001). In particular, the inhibitory abilities (Amieva et al., 2004), the attentional (Perry and Hodges, 1999) and the visuospatial functions (Cronin-Golomb and Amick, 2001) would be specifically compromised.

In patients with the AD, the attentional skills needed to resolve complex tasks would be impaired, such as divided attention, the ability to effectively disengage and shift attention (Perry and Hodges, 1999) and sustained attention (Berardi et al., 2005). Moreover, about the visuospatial functions the constructive praxia, visual-perceptive, and visual orienting abilities would seem to be damaged (Cronin-Golomb et al., 2007). When these cognitive deficits interfere with the performance of daily life activities, the patient can react to his/her cognitive impairment with mood swings, irritability and apathy. All these aspects outline the characteristic clinical profile associated with Alzheimer's disease.

Considering the relevance that the AD has on the life of patients affected by this disease, it is essential to understand the specific alterations involving the executive functioning thoroughly. With this purpose, recently, many researchers have focused on the use of experimental paradigms aimed at analyzing the deficits of executive functions in individuals affected by dementia (Sgaramella et al., 2001; Bullock and Lane, 2007; Cronin-Golomb et al., 2007; Ramanan et al., 2017). These studies highlighted how the various executive functions (Miyake et al., 2000) are differently affected by AD depending on the stage of the disease and by the personal characteristics of the patients.

## Cognitive Tasks and Assessment of Cognitive Functions in Alzheimer's Disease

Different experimental paradigms were used to evaluate executive functions in the AD. (Perry and Hodges, 1999) Given the heterogeneity of these paradigms and the vastness of studies aimed at investigating executive performance in patients with the AD, the objective of this review is to analyse the researches that address this issue through four specific behavioral tasks: Stroop Task, Wisconsin Card Sorting Test, Flanker Task, and Go/No-Go Task. These tasks were more commonly used to evaluate executive performance (Diamond, 2013).

Stroop Task (Stroop, 1935) is one of the most used paradigms for the study of executive functions. In particular, through the use of incongruent stimuli, it evaluates the management of the conflict and the inhibitory control of automatic responses. In the standard version of the Stroop Task, the stimuli are words written with colored inks. There are congruent (the word RED written in red ink) or incongruent (the word RED written in green ink) trials; the participant's goal is to respond by referring to the color of the ink ignoring the meaning of the word. In this way, two alternative and incompatible responses (color vs. word) are elicited, one of which is more spontaneous than the other (reading of the word vs. ink color denomination).

The Wisconsin Card Sorting Test (WCST) (Milner, 1963) is aimed to evaluate abstract reasoning, and cognitive flexibility understood as the ability to change one's strategies in response to environmental contingencies (Berg, 1948; Grant and Berg, 1948; Luria, 1973; Shallice, 1982). The WCST consists of four stimulus cards and two sets of 64 response cards. The cards vary in color, shape and number of elements represented. The test includes some ambiguous stimuli, and the pairing criteria vary according to a standardized order (Color, Form, Number). The task requires identifying the correct criterion with which to order the response cards to the stimulus cards; for each card placed by the participant, the experimenter provides feedback on the correctness of the performance. Based on the feedback from the experimenter, the participant can modify his/her behavior by identifying the appropriate strategy.

The Flanker Task (Eriksen and Eriksen, 1974) measures selective attention and the ability to control conflictual information. The task requires discriminating the central target stimulus between a series of lateral distractors (flanker). There are three types of conditions: the congruent trials, in which the target stimulus and the flankers have the same characteristics and required the same response; the incongruent trials, in which the target has different features with respect to the distractors, requiring an opposite response that generates conflict; finally, the neutral condition, in which the distractor is not confused with the targets presented in the task, and it does not cause conflict. The flanker effect (also called conflict or congruence effect) reveals the difficulty in ignoring the distractors due to the ambiguity of the stimuli used (Cohen and Shoup, 1997).

The Go/No-Go Task assesses sustained attention (vigilance) and impulsivity and allows obtaining information related to motor-type inhibitory control (Zahn et al., 1980, 1991; McGaughy and Sarter, 1995). The task consists in the presentation of a stimulus that requires a response from the participant (Go stimulus), and another stimulus for which the participant must, instead, inhibit any response (No-Go stimulus). A high percentage of errors indicates a difficulty in behavioral inhibition. Also, in this case, there are different versions of the task to investigate the inhibitory aspects and the influences of this ability from other variables, such as emotions (Schulz et al., 2007).

## Aims

The central aim of this review is to analyse the sensitivity of four experimental paradigms (Stroop Task, Flanker Task, Wisconsin Card Sorting Test and Go/No-Go Task) in the study of executive functions in elderly subjects suffering from Alzheimer's dementia, in order to be able to consider and define the applicability and usefulness of these paradigms. Moreover, another objective of this work is to verify how the executive functioning in the AD is compromised concerning the normal operation of healthy elderly, with the aim of understanding how and where the cognitive impairment associated with dementia intervenes in a more evident way.

## Systematic Review

The systematic review was conducted using the PRISMA method (Moher et al., 2009), but without recording the protocol. This review considered all the works that investigated the executive functioning through the use of the cognitive tasks defined in the introduction.

Most of the considered studies refer to the diagnostic criteria of Alzheimer Disease of the National Institute of Neurological and Communications Disorders and Stroke and the Alzheimer's Disease and Related Disorders Association (NINCDS-ADRDA) (McKhann et al., 1984) for the classification of the AD. However, we considered also the studies that used the diagnosis criteria of DSM (American Psychiatric Association, 1980, 1994), the Cambridge Diagnostic Examination of Elderly (CAMDEX) (Roth et al., 1986) or National Institutes of Health and the Alzheimer's Association published revised guidelines (NIA-AA) (McKhann et al., 2011).

## Method

The study was the result of systematic research in the PubMed, PsycArticles, and PsycINFO databases. The following keywords were used for the search: Stroop Task, Wisconsin Card Sorting Test, Go/No-Go Task, Flanker Task, and Alzheimer. Thespecific scripts are presented in Table 1.

**Table 1:** Scripts used in the systematic research.

	**Script**
Alzheimer and Stroop Task	(“Alzheimer disease”[MeSH Terms] OR (“Alzheimer”[All Fields] AND “disease”[All Fields]) OR “Alzheimer disease”[All Fields] OR “Alzheimer”[All Fields]) AND (“Stroop test”[MeSH Terms] OR (“Stroop”[All Fields] AND “test”[All Fields]) OR “Stroop test”[All Fields] OR (“Stroop”[All Fields] AND “task”[All Fields]) OR “Stroop task”[All Fields]).
Alzheimer and Flanker Task	(“Alzheimer disease”[MeSH Terms] OR (“Alzheimer”[All Fields] AND “disease”[All Fields]) OR “Alzheimer disease”[All Fields] OR “Alzheimer”[All Fields]) AND Flanker[All Fields] AND Task[All Fields].
Alzheimer and Go/No-Go	(“Alzheimer disease”[MeSH Terms] OR (“Alzheimer”[All Fields] AND “disease”[All Fields]) OR “Alzheimer disease”[All Fields] OR “Alzheimer”[All Fields]) AND Go/No-Go[All Fields] AND Task[All Fields].
Alzheimer and Wisconsin Card Sorting Test	(“Alzheimer disease”[MeSH Terms] OR (“Alzheimer”[All Fields] AND “disease”[All Fields]) OR “Alzheimer disease”[All Fields] OR “Alzheimer”[All Fields]) AND (“Wisconsin card sorting test”[MeSH Terms] OR (“Wisconsin”[All Fields] AND “card”[All Fields] AND “sorting”[All Fields] AND “test”[All Fields]) OR “Wisconsin card sorting test”[All Fields]).

All articles published on the topic up to the date of 1 July 2018 have been taken into account.

Two researchers performed the research independently, and the results were compared. The disagreements have been resolved with consensus methods. In case of lack of consensus among the researchers, a supervisor was used.

For the selection of the articles the following inclusion criteria were used: publications on “Peer Review-Journals”; use of the Stroop Task, the Flanker Task, the Wisconsin Card Sorting Test or, the Go/No-Go Task; the presence of a control group of healthy elderly.

The exclusion criteria were the following: (a) studies that did not present all the data useful for a critical analysis of the results; (b) the use of versions of the tasks that considered the emotional components of executive functions; (c) studies with methodological bias (for example with unspecified inclusion/exclusion criteria); (d) studies comparing the AD group with groups affected by other types of dementia or MCI without a healthy elderly group; single cases.

The initial results produced 858 articles. After the elimination of duplicates and irrelevant papers, by the title and abstract reading, 83 articles were read.

**Figure 1:**
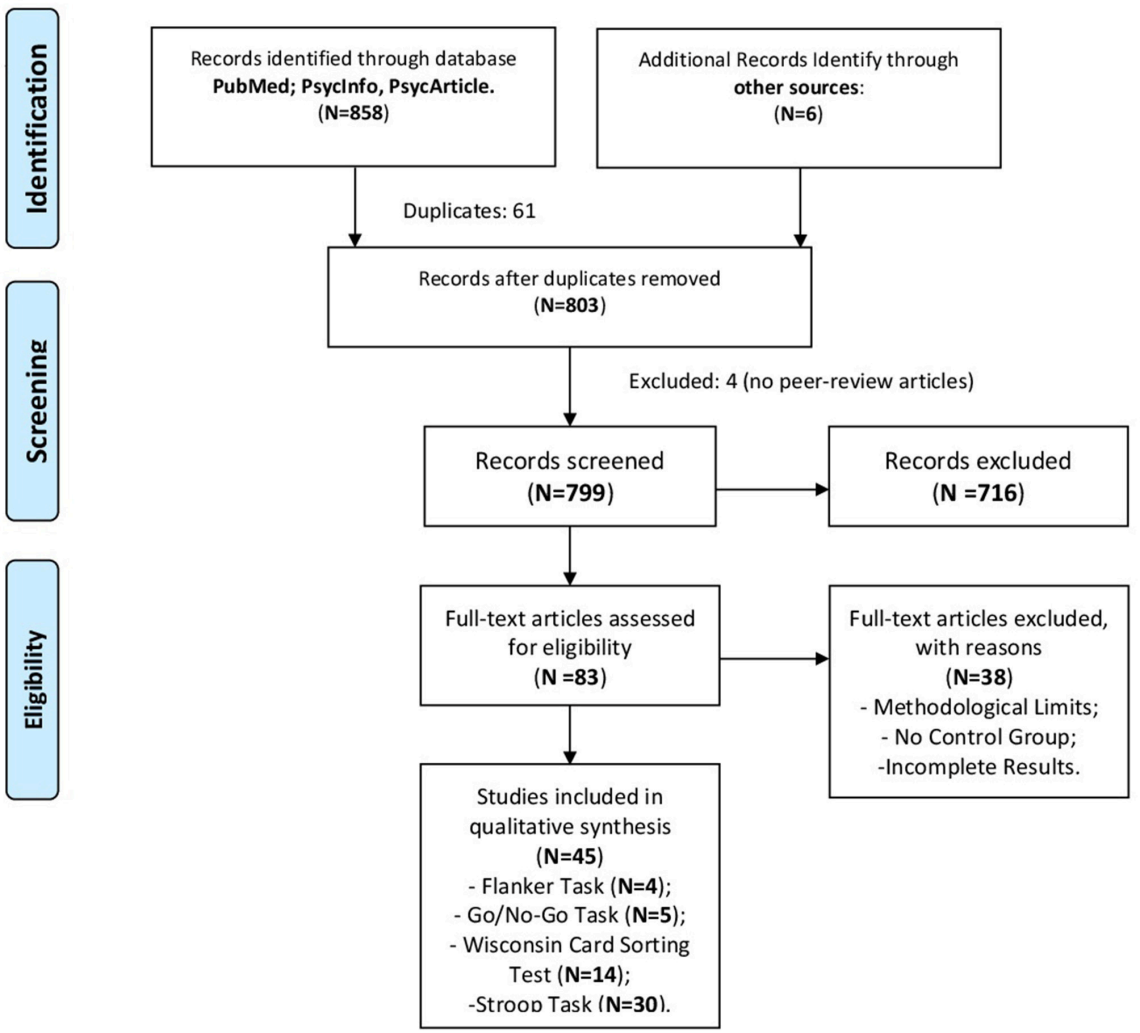
The flowchart of the studies selection.

At the end of the revision work, 45 articles were included in the review. The flowchart presented in Figure 1 shows the selection of the studies.

The 45 selected articles have been categorized concerning the single paradigm used. Studies that used more experimental tasks were discussed in the different paragraphs concerning the results of each specific task. According to PICOS (Moher et al., 2009), information about participants, control groups, methods and results have been extracted. These data are presented in the different behavioral task tables.

## Results

### Stroop Task

The systematic research has allowed the identification of 30 studies (see Table 2) that used different versions of the Stroop Task to evaluate inhibitory control and selective attention in patients with Alzheimer's disease.

**Table 2:** Some characteristics of the studies that have used the Stroop Task to assess executive dysfunction in patients with Alzheimer's disease.

**Studies**	**Participant N (mean age ±*SD*)**	**MMSEa**	**Diagnostic criteria for Adb**	**Stroop task typologies**	**Differences between AD group and CG group**		
					**Accuracy**	**Reaction time**	**Other indices**
Koss et al., 1984	CG = 11 (66.0) [5M;6F]	DRS4	DSM-III	Three conditions: Color (XXXs strings), Word (neutral word), Color-Word (naming the ink of the color word).	AD < CG	AD>CG	–
	Mild AD = 9 (62.3) [5M;4F]	Mild AD = 111.6					
	Moderate AD = 6 (67.6) [4M;2F]	Moderate AD = 73					
	MCI = 6 (60.7) [6M;0F]	MCI = 110					
Fisher et al., 1990	CG = 36 (72.9 ± 8.3) [13M;23F]	BDSe CG = 1.5 ± 6.1	NINCDS-ADRDA6	Three conditions: Color (cross), Word (Black Ink), Color-Word (Golden and Freshwater, 1978).	–	–	Interference Score: AD worse than AD
	AD = 36 (70.1 ± 8.9) [14M;22F]	AD = 15.5 ± 6.1					
Spieler et al., 1996	Older CG = 50 (85.6 ± 3.4)	CDR	NINCDS-ADRDA	Two conditions: Neutral word, Xs or symbols; Color-Word.	AD < CG	AD>CG	Facilitator Effect [(RT congruent-RT incongruent)/RT neutral]: AD>CG
	Young CG = 25 (70.5 ± 6.7)						Interference Effects [RT incongruent-RT neutral]: AD = CG
	Mild AD = 25 (73.2 ± 6.5)						
	Severe AD = 40 (73.7 ± 8.8)						
Amieva et al., 2002	CG = 28 (75.2 ± 6.6)	CG = 27.6 ± 1.8	NINCDS-ADRDA	Three Conditions: Color; Word; Color-Word.	AD < CG	AD>CG	Interference Ratio [RTon C-W task/RT on C task]: AD>CG
	Mild AD = 28 (75.8 ± 6.1)	Mild AD = 24.6 ± 1.9					
Bondi et al., 2002	CG = 51 (73.40 ± 7.43) [20M;31F]	DRS	NINCDS-ADRDA	Three conditions: Color (cross); Word (Black Ink); Color-Word (Golden and Freshwater, 1978).	AD < CG	AD>CG	–
	AD = 59 [31M;28F]	Very Mild = 119–139					
	Very mild AD = 22 (71.7 ± 6.97)	Mild = 106–118					
	Mild AD = 25 (73.5 ± 5.76)	Moderate = 90–105					
	Moderate AD = 12 (72.1 ± 8.41)						
Amieva et al., 2004	CG-I = 22 (74.0 ± 4.7) [5M;17F]	CG-I = 27.9 ± 1.7	NINCDS-ADRDA	Four conditions: *Interference* (word-ink, naming the ink); *Reading* (word in black); *Color* (color patches) *Reverse Stroop Task* (reading the word).	AD < CG (in all condition)	AD>CG (in all condition)	–
	AD-I = 22 (74.0 ± 5.1) [5M;17F]	AD-I = 21.1 ±3.0					
	CG-R = 22 (74.9 ± 6.4) [6M;16F]	CG-R = 27.5 ± 1.7					
	AD-R = 22 (74.8 ± 6.3) [6M;16F]	AD-R = 21.4 ± 2.4					
Levinoff et al., 2004	CG = 23 (73.0 ± 6.1) [10M;13F]	CG = 28.8 ± 1.0	NINCDS-ADRDA	Two subtests: Color (dots), Color-Word (Stroop, 1935).	–	AD>RT	Interference Effect RT:
	AD = 30 (74.1 ± 8.3) [16M;14F]	AD = 22.4 ± 3.1					[RT C-W- RT C]: AD>CG
Belleville et al., 2006	CG = 12 (72.7 ± 4.6) [6M;6F]	CG = 28.2 ± 1.1	NINCDS-ADRDA	Three conditions; Word Condition (name of color in black ink), Color Condition (xxx in color ink), Interference Condition (color word in different color ink). Number of correct responses in 45 s (Golden, 1976).	AD < CG	–	Inhibition Score [Interference/ (Word+Color)/2]: AD>CG
	Young CG = 12 (22 ± 3.2) [4M;8F]	Young CG = N/A					
	AD = 12 (72.5 ± 5.9) [4M;8F]	AD = 22.9 ± 2.0					
Duong et al., 2006	CG = 60 (74.38 ± 5.74)	CG = 29.12 ± 0.97	NINCDS-ADRDA	*Classic Stroop Task*. Three conditions: dot (4 dots x 6 lines in four different colors), word (4 words x 6 lines, word in the same color as before), Color-Word (4 color words in incongruent ink x 6 line in the same color as before).	Stroop Task: AD < CG (in all conditions	Stroop Task: AD>CG (in all conditions)	Stroop Task: Interference dot-baseline (ACC e RT). AD worse than CG.
	AD = 36 (73.62 ± 8.94)	AD = 22.08 ± 3.76		*Stroop Pictures Naming*: Picture of animals and letter strings in the drawing. Four conditions: *Congruent* (letter string is the name of the animals drawing), *Incongruent/same item* (letter string is the name of a different animals in the task), *Incongruent/different item* (letter string is the name of an animal not presents in the task), *Neutral* (xxx on the animal drawing).	Stroop Pictures Naming:	Stroop Pictures Naming:	Interference word-baseline (ACC e RT). AD worse than CG.
	MCI = 61 (74.68 ± 6.48)	MCI = 27.20 ± 2.15			AD < CG	AD>CG	Stroop Pictures Naming: Facilitation: AD = CG (in RT and n° of errors)
							Inhibition different AD = CG (RT), AD>CG (n° of errors)
							Inhibition similar AD = CG (RT), AD>CG (n° of errors)
Stokholm et al., 2006	CG = 32 (74.3 ± 4.2)	CG = 29.3 ± 0.9	NINCDS-ADRDA	Color-Word condition (Stroop, 1935).	AD < CG	AD>CG	
	AD = 36 (76 ± 5.6)	AD = 25 ± 1.5					
Belleville et al., 2008	CG(AD) = 19 (72.42 ± 8.31) [4M;15F]	CG(AD) = 28.74 ± 0.94	NINCDS-ADRDA	Stroop Victoria (Spreen and Strauss, 1998).	Incongruent: AD < CG		
	AD = 19 (73.42 ± 9.18) [9M;10F]	AD = 24.65 ± 3.60		Short version (24 items for each condition), three conditions: Color, Neutral Word, Color-Word.		
	CG(MCI) = 25 (66.12 ± 10.09) [5M;20F]	CG(MCI) = 28.88 ± 0.99				
	MCI = 25 (64.76 ± 10.83) [11M;14F]	MCI = 28.36 ± 1.98				
Bracco et al., 2007	CG = 13 (70.46 ± 6.33) [5M;8F]	CG = 29.38 ± 0.87	–	Three conditions: Word, Color, Color-Word (Stroop, 1935).	–	Interference (C-W) AD>CG	.
	AD = 50 (73.6 ± 7.1) [10M;40F]	AD = 21.3 ± 3.3					
	Very Mild AD = 22 (75.1 ± 6.86) [6M;16F]	Very Mild AD = 23.7 ± 2.2					
	Mild AD = 28 (72.4 ± 7.1) [4M;24F]	Mild AD = 19.5 ± 2.8					
Collette et al., 2007	CG = 28 (70.6 ± 6.8) [13M;15F]	DRS	NINCDS-ADRDA	Two sets: Color, Color-Word (interference, ink and word: incongruent color); naming the ink of the color word (Stroop, 1935).	Color naming: CG = AD	Color naming: AD>CG	Interference ratio [RT Interference Condition/ RT Color Condition]: AD = CG
	AD = 25 (72.5 ± 5.8) [18M;17F]				Stroop: Task: AD < CG	Stroop: AD>CG	Interference differential score [RT Interference Condition-RT Color Condition]:AD>CG
	FTD = 13 (65.7 ± 7.5) [5M;8F]						
Doninger and Bylsma, 2007	CG = 30 (83.78 ± 7.81) [6M;24F]	GC = 29 ± 0.87	NINCDS-ADRDA	*Emotional Stroop Task*: emotive word in different color. Naming the color.			
	Mild AD = 24 (85.34 ± 6.09) [7M;17F]	Mild AD = 25.25 ± 1.80		*Color-Word Stroop* (Golden and Freshwater, 1978).			
	Moderate AD = 21 (79.75 ± 9.89) [10M;11F]	Moderate AD = 16.05 ± 3.94					
Belleville et al., 2008	CG = 25 (72.8 ± 7.6) [14M;11F]	CG-AD = 28.69 ± 0.8	NINCDS-ADRDA	Stroop Victoria (Regard, 1981).	–	In the C-W condition: AD>CG	–
	AD = 13 (73.2 ± 8.1) [6M;7F]	AD = 24.85 ± 4	DSM-IV	Short version (24 items for each condition), three conditions: Color, Neutral Word, Color-Word			
	MCI = 20 (66.3 ± 10.9) [8M;12F]	MCI = 28.15 ± 2.1					
Li et al., 2009	CG = 9 (65.2 ± 7.2) [4M;5F]	CG = 28.8 ± 0.9	DSM-IV	Three color names with three color inks (Leung et al., 2000).	AD < CG	AD>CG	–
	AD = 10 (65.8 ± 6.1) [5M;5F]	AD = 16.7 ± 2.6					
	MCI = 9 (63.4 ± 4.6) [5M;4F]	MCI = 26.4 ± 4.2					
Duchek et al., 2009	CG Older = 220 (71.75 ± 8.31)	CG Older = 29.09 ± 1.17	NINCDS-ADRDA	Two conditions: color word (*incongruent*) and neutral word (*congruent*) with ink color. Naming the ink of the word.	–	–	Congruent Effect RT [RT Congr- RT Incongr]: AD>CG
	Very Mild AD = 71 (75.25 ± 7.68)^*^	Very Mild AD = 26.95 ± 2.36					Congruent Effect ACC [Error Congr- Error Incongr]: AD>CG
Bélanger et al., 2010	CG Young = 20 (23.9 ± 4.7)	CG Young	NINCDS-ADRDA	Stroop version 1) Neutral Condition and Incongruent condition (two different blocks).	AD < CG	AD>CG	–
	CG Older = 20 (71.10 ± 7.5)	n.a		Stroop version 2 Congruent Condition and Incongruent Condition (mixed block: 75% congruent, 25% incongruent).			
	AD = 11 (75 ± 6.4)	CG Older 28.8 ± 1.4					
	MCI = 20 (72.7 ± 6.8)	AD 23.4 ± 3.7					
		MCI 27.4 ± 2.1					
Hutchison et al., 2010	Older CG = 64 (77.24 ± 9.80)	Older CG = 29.19	CDR	*Stroop Switch Task* [pc version; stimuli from (Spieler et al., 1996)]. Color word or neutral word. No congruent trial. Participant switching the responding (or color of the ink or reading of the word).	AD < CG	AD>CG	–
	Young CG = 30 (20.8 ± 1.5)	AD = 28.22					
	AD = 32 (78.78 ± 5.89)					
McGuinness et al., 2010	CG = 28 (70.2 ± 7.9)	CG >28	NINCDS-ADRDA	Three conditions: Color (cross), Word (Black Ink), Color-Word (incongruent). Naming the ink of the word (Golden and Freshwater, 1978).	Color: AD < CG	–	–
	AD = 75 (77.7 ± 6.9) [24M;52F]	AD and VaD >12	NINDS- AIREN for Vascular Dementia		Word: AD < CG		
	VaD = 46 (75.9 ± 7.8) [22M;24F]				Color-Word: AD < CG		
Tse et al., 2010	CG = 246 (71.77 ± 7.71)	CDR 0.5 = Very Mild AD	NINCDS-ADRDA	One condition: Color-Word, *congruent* (color and word are the same), *incongruent* (color and word are different), *neutral* (words are not color name). Naming the color ink.	.	.	Stroop-Effect [%errors incongruent trials- % errors congruent trials] = AD>CG
	Young CG = 32 (20.31 ± 1.12)						Stroop-Effect [RT incongruent trials- RT congruent trials] = AD = CG
	Very Mild AD = 74 (75.82 ± 781)^*^						
Coubard et al., 2011	CG Older = 17 (77.65 ± 7.72) [10M;7F]	–	–	Three conditions: Color (cross), Word (black ink), Color-Word (*incongruent* ink: suppressing the reading, naming the ink) (Godefroy, 2008).	–	–	Interference ratio n. correct responses in C-W condition/n. correct responses in C condition: AD = CG.
	CG Young = 18 (25.36 ± 2.78) [8M;10F]						Interference error rate [% errors C-W condition- %errors C condition]: AD = CG
	AD = 17 (78.68 ± 6.15) [3M;14F]						
Li et al., 2011	CG = 8 (66) [3M;5F]	CG = 28.7	–	Three conditions: Word, Color, Color-Word (Stroop, 1935).	AD < CG		Interference Effect: AD>CG
	AD = 6 (68) [3M;3F]	Demented = 20.4					
	SVD = 6 (66) [4M;2F]						
Yun et al., 2011	CG = 54 (70.4 ± 5.7)[66.7%F]	CG = 26.8 ± 2.3	NINCDS-ADRDA	Three conditions: Word, Color, Color-Word. Number of responses in 45 s (Golden and Freshwater, 2002).	–	–	Interference Indices: AD worse than CG.
	AD = 136 (70.2 ± 8.4) [75%F]	AD = 16.6 ± 5.1	DSM-IV				
Stawarczyk et al., 2012	Study2	MMSE > 21	NINCDS-ADRDA	Three sets of stimuli: color string (neutral; %%%), congruent stimuli (ink and word same color), incongruent stimuli (ink and word different color); participant were asked to say aloud quickly and accurately as possible the name of the ink. Four conditions: Congruent, Incongruent, Positively Primed Congruent trials, Negatively Primed Incongruent Trials (Hogge et al., 2008).	Neutral: AD = CG	Neutral: AD = CG	Interferent effect RT [RT Neutral Condition- RT Interference Condition]: AD = CG
	CG = 16 (76.6 ± 10.6) [7M;9F]				Interferent: AD < CG	Interferent: AD = CG	Interferent effect ACC [ACC Neutral Condition- ACC Interference Condition]: AD>CG
	MildAD = 16 (75.3 ± 10.3) [7M;9F]						Negative Priming Effect [Negatively Primed Incongruent Trials RT- Positively Congruent Trials RT]
Chen et al., 2013	CG = 100 (75.4 ± 7.3) [68M;32F]	CG = 28.4 ± 1.7	NINCDS-ADRDA	Correct responses in 120 s of the task.	AD < CG	–	–
	aMCI = 120 (78.2 ± 7.7) [82M;38F]	aMCI = 26.6 ± 1.4	Petersen et al. (1999) Criteria For MCI				
	AD = 126 (78.9 ± 5.5)	AD = 20.2 ± 3.6					
	[88M;38F]						
El Haj et al., 2013	CG Young = 18 (21.78 ± 3.56) [6M;12F]	CG Young n.a.	NINCDS-ADRDA	[pc-version]. Word, Color, Color-Word (*interference:* only incongruent trials).	–	–	Interference Effect: [Interference TR- (mean Color and Word TR)]: AD >CG
	CG Older = 18 (73.28 ± 6.35) [6M;12F]	CG Older = 28.28 ± 1.32	in the mild stage of severity dementia				
	AD = 18 (76.11 ± 5.92) [5M;13F]	AD = 23.23 ± 1.59					
Peltsch et al., 2014	CG = 72 (73 ± 6) [22M;50F]	CG = 29 ± 1	NINCDS- ADRDA	Two conditions: Color and Color-word.	No Cov: AD>CG	–	–
	aMCI = 22 (76 ± 8) [10M;12F]	aMCI = 27 ± 2			Cov: AD < CG		
	Mild AD = 24 (76 ± 8) [9M;15F]	Mild AD = 27 ± 2					
Sánchez-Benavides et al., 2014	CG = 356 (64.9 ± 9.3) [144M;212F]	CG = 28.7 ± 1.5	NINCDS- ADRDA	Word, Color, Color-Word.	Color: AD < CG		
	MCI = 79 (72.8 ± 6.5) [34M;45F]	MCI = 25.7 ± 2.2			Word: AD < CG		
	AD = 100 (74.4 ± 7.5)	AD = 20.2 ± 4.0			Color-Word: AD < CG		
	[35M;65F]						
Huang et al., 2017	CG = 31 (76.5 ± 5.9) [45.2%F]	CG = 27.0 ± 1.2	NINCDS- ADRDA	One condition: *incongruent* (naming the ink of the color word) (Golden and Freshwater, 2002).	AD < CG	–	–
	Mild AD = 31 (78.9 ± 6.3) [64.5%F]	Mild AD = 21.2 ± 3.2	DSM-IV-TR CDR				

* *There is a significant difference between groups. MMSE, Mini Mental State Examination; AD, Alzheimer Disease; CG, Control Group; DRS, Dementia Rating Scale; BDS, Blessed Dementia Scale; NINCDS-ADRDA, National Institute of Neurological and Communicative Disorders and Stroke and the Alzheimer's Disease and Related Disorders Association; RT, Reaction Time; C-W, Naming incongruent ink color on the Stroop Task (Interference task); C, Naming Color Task on the Stroop Task; CG-I, Control Group Interference task; AD-I, Alzheimer Disease Interference task; CG-R, Control Group Reverse task; AD-R, Alzheimer Disease Reverse task; N/A, Data Not Available; MCI, Mild Cognitive Impairment; ACC, Accuracy; FTD, Frontotemporal Dementia; CDR, Clinical Dementia Rating Scale; VaD, Vascular Dementia; SVD, Subcortical Vascular Dementia*.

Within the 30 studies, only five of these did not use the diagnostic criteria of the National Institute of Neurological and Communicative Disorders and the Alzheimer Disease Association (McKhann et al., 1984), in particular, Koss et al. (1984) used the criteria for the diagnosis of AD diagnosis of DSM-III, medical observation and scores obtained at the Dementia Rating Scale (DRS) (Mattis, 1988); Li and collaborators (Li et al., 2009, 2011), have used the diagnostic criteria of the DSM-IV; (Fisher et al., 1990)used the Blessed Dementia Scale (Blessed et al., 1968) and Hutchison and colleagues (Hutchison et al., 2010) used the Clinical Dementia Rating Scale (CDR) (Morris, 1993).

The patients with the AD and their control groups included all participants over 65 years old, who differed from each other for the scores obtained at the Mini-Mental State Examination (MMSE) (Folstein et al., 1975) or the DRS for the evaluation of the severity of dementia. Only in the study of Tse and colleagues (Tse et al., 2010), there was a significant difference between the age of patients with the AD and the healthy elderly group (patients with AD were older than people of the control group).

Compared to the Stroop Task analysis, all authors evaluated at least one of the following dependent variables: accuracy of responses, reaction times and interference effect (or Stroop effect).

In most of the studies, the analysis of accuracy showed worse performance in the patients with AD compared to healthy elderly (Koss et al., 1984; Spieler et al., 1996; Amieva et al., 2002, 2004; Bondi et al., 2002; Belleville et al., 2006, 2008; Duong et al., 2006; Stokholm et al., 2006; Collette et al., 2007; Doninger and Bylsma, 2007; Bélanger et al., 2010; Hutchison et al., 2010; McGuinness et al., 2010; Tse et al., 2010; Li et al., 2011; Stawarczyk et al., 2012; Chen et al., 2013; Peltsch et al., 2014; Sánchez-Benavides et al., 2014; Huang et al., 2017).

Furthermore, in all studies that also evaluated reaction times, it was found that patients with Alzheimer's disease are generally slower than healthy elderly people; (Koss et al., 1984; Spieler et al., 1996; Bondi et al., 2002; Amieva et al., 2004; Levinoff et al., 2004; Duong et al., 2006; Stokholm et al., 2006; Collette et al., 2007; Li et al., 2009; Bélanger et al., 2010; Hutchison et al., 2010; Tse et al., 2010) this result was not observed by Stawarczyk et al. (2012) that found similar reaction times in the two groups.

In the studies identified by systematic research, several indices were used to detect deficits in the inhibitory control through the Stroop Task. The most commonly used is the Interference Effect Index, generally calculated through the ratio or subtraction of the performance values (on reaction times or Accuracy) between the Neutral condition (Color or Word) and the interference condition (Color-Word). The results showed a higher interference effect in the patients with AD groups than in the healthy elderly people (Fisher et al., 1990; Levinoff et al., 2004; Duong et al., 2006; Collette et al., 2007; Li et al., 2009; Tse et al., 2010; Yun et al., 2011; Stawarczyk et al., 2012). The interference effect can also be considered as a measure of inhibition, and patients with AD would exhibit lower inhibitory ability than healthy participants (Amieva et al., 2004; Belleville et al., 2006). In addition, some studies have evaluated additional indices and effects: among these, Spieler and colleagues (Spieler et al., 1996) have identified a higher facilitator effect in patients with AD in the congruent trials of the Stroop Task; while Amieva and collaborators (Amieva et al., 2004) have shown a higher difficulty of patients with Alzheimer's disease in inhibiting previously imposed rules.

### Wisconsin Card Sorting Test

The systematic research allowed highlighting 14 studies (see Table 3) that used the WCST to investigate the executive function in patients with Alzheimer's disease. For this purpose, different versions of the test were used.

**Table 3:** Some characteristics of the studies that have used the Wisconsin Card Sorting Test to assess executive dysfunction in patients with Alzheimer's disease.

**Studies**	**Participant N (mean age ±SD)**	**MMSE**	**Diagnostic criteria for AD**	**WCST typologies**	**Differences between AD group and CG group**
Hart et al., 1988	CG = 18 (70.6 ± 5.2) [78%F]	CDR	Non-specific criteria	mWCST (modified version: 72 cards): no ambiguous cards; after six correct responses there was the category shifting; no information about the shift in sorting (Nelson, 1976).	Achieved Categories: AD < CG
	DEP = 17 (69.3 ± 6.2) [65%F]				Number of Errors: AD>CG
	Mild AD = 18 (71.6 ± 6.4) [76%F]				Perseverative Errors: AD>CG
	Moderate AD = 16 (72.1 ± 6.8)				
Bhutani et al., 1992	CG = 12 (63.7 ± 8.27)	CG = 28. 8 ± 1.64	CAMBEX	WCST (original version: 128 cards) (Milner, 1963).	Achieved Categories: AD = CG
	MinimallyAD = 11 (71.5 ± 7.97)	Minimally AD = 25.5 ± 3.69			Perseverative Errors: AD = CG
	Mildly AD = 6 (74 ± 12.88)	Mildly AD = 18.7 ± 2.73			Non-Perseverative Errors: AD = CG
	Moderately AD = 8 (79.7 ± 6.45)	Moderately AD = 11.81 ± 3.95			
Bondi et al., 1993	CG = 75 (71.1 ± 7.6)[48F]	CG = 28.9 ± 1.2	NINCDS-ADRDA	mWCST (modified version: 48 cards): no ambiguous cards; after six correct responses subject was informed of a shift in sorting principles (Nelson, 1976).	Achieved Categories: AD < CG
	Mild AD = 23 (72.7 ± 5.9) [12F]	Mild AD = 23.9 ± 2.3			Perseverative Errors: AD>CG
	Moderate AD = 33 (72.3 ± 5.9) [20F]	Moderate AD = 21.2 ± 2.9			Non-Perseverative Errors: AD>CG
	Severe AD = 31 (71.8 ± 7.9) [14F]	Severe AD = 17.8 ± 3.7			
Paulsen et al., 1995	CG MiddleAge = 20 (49.7 ± 13.9) [10M;10F]	DRS Middle Age CG = 141.3 ± 2.7	NINCDS-ADRDA	mWCST (modified version: 48 cards): no ambiguous cards; after six correct responses subject was informed of a shift in sorting principles (Nelson, 1976).	Achieved Categories: AD < CG
	Elderly CG = 20 (69.7 ± 8.2) [10M;10F] AD = 20 (70.0 ± 6.9) [10M;10]	Elderly CG = 140.1 ± 2.3			Correct Responses: AD < CG
	HD = 20 (69.7 ± 8.2) [12M;8F]	AD = 121.4 ± 8.6			Perseverative Errors: AD>CG
		HD = 120.3 ± 9.6			
Paolo et al., 1996	CG = 35 (71.34 ± 7.73) [22M;13F]	DRS	NINCDS-ADRDA	WCST-64: classical WCST rules, but in a short version (Heaton, 1981).	Achieved Categories, Trials to complete the 1 st category, Number of Errors, Perseverative Responses, Perseverative Errors, Non-Perseverative Errors, Percent Conceptual Level Response: AD worse than CG.
	PDN = 35 (70.51 ± 5.55) [22M;13F]				Failure to Maintain Set: AD = CG.
	PDD = 35 (71.77 ± 6.31) [22M;13F]				
	AD = 35 (71.06 ± 5.75) [22M;13F]				
Tei et al., 1997	CG = 30 (65.1 ± 8.2)	CG = 29.1 ± 0.6	NINCDS-ADRDA MSI = Hacinski ischemic score >7	WCST (original version: 128 cards) (Milner, 1963).	Achieved Categories: AD < CG
	AD = 22 (67.3 ± 9.1)	AD = 23.6 ± 1.4			Perseverative Errors: AD >CG
	MCI = 22 (68.6 ± 7.3)	MCI = 24.6 ± 2.0			
Nagahama et al., 2003	CG = 22 (70.8 ± 9.1)	CG = 29.1 ± 0.8	NINCDS- ADRDA	mWCST (computer version) 48 cards no ambiguous cards; participant was informed of the three possible categories before testing; after six correct responses there was the category shifting; no information about the shift in sorting (Nelson, 1976; Jenkins and Parsons, 1978).	Total Errors, Trials to complete the 1^st^category, Perseverative Errors: AD>CG;
	AD = 54 (74.2 ± 5.1)	AD = 20.8 ± 3.3	DSM-III-R		Achieved Categories, Non-perseverative Errors, Conceptual level responses: AD < CG.
	MCI = 17 (72.8 ± 5.4)	MCI = 26.4 ± 2.0			
Stokholm et al., 2006	CG = 32 (74.3 ± 4.2)	CG = 29.3 ± 0.9	NINCDS- ADRDA	mWCST (modified version: 48 cards): no ambiguous cards; after six correct responses subject was informed of a shift in sorting principles (Nelson, 1976).	Achieved Categories: AD>CG
	AD = 36 (76 ± 5.6)	AD = 25 ± 1.5			Number of Errors: AD = CG
					Perseverative Errors: AD = CG
Kugo et al., 2007	CG = 25 (63.7 ± 2.4) [12M;13F]	CG = 28.2 ± 1.9	NINCDS- ADRDA	KWCST (Keio version): participant was informed about the presences of three categories (Abe et al., 2004).	Achieved Categories: AD>CG
	AD = 58 (75.3 ± 7.8) [14M;44F]	AD = 19.3 ± 4.1	CDR		Perseverative Errors: AD = CG
	VaD = 24 (75.1 ± 9.3) [10M;14F]	VaD = 20.7 ± 4.7			
	FTD = 23 (64.7 ± 9.5) [9M;14F]	FTD = 19.6 ± 5.9			
Chen et al., 2009	CG = 16 (69 ± 8.4) [9M;7F]	CDR	NINCDS-ADRDA	mWCST (modified version: 48 cards): no ambiguous cards; after six correct responses subject was informed of a shift in sorting principles (Nelson, 1976).	Achieved Categories: AD < CG
	Early AD = 11 (76.7 ± 8.5) [7M;4F]		Mayo Clinic Criteria for aMCI		Perseverative Errors: AD>CG
	aMCI = 13 (73.2 ± 9.3) [8M;5F]				
Chiu et al., 2014	CG = 30 (64.4 ± 9.5) [13M;17F]	CG = 28.8 ± 1.6	NIA-AA	WCST (original version: 128 cards) (Milner, 1963).	Achieved Categories: AD < CG
	Early AD = 10 (69.3 ± 9.4) [4M;6F]	Early AD = 22.7 ± 3.6			Perseverative Errors: AD = CG
	MCI = 20 (71.2 ± 9.7) [9M;11F]	MCI = 26.3 ±2.7			
Peltsch et al., 2014	CG = 72 (73 ± 6) [22M;50F]	CG = 29 ± 1	NINCDS-ADRDA	WCST (original version: 128 cards) (Milner, 1963).	% Errors: AD>CG
	aMCI = 22 (76 ± 8) [10M;12F]	aMCI = 27 ± 2			
	Mild AD = 24 (76 ± 8) [9M;15F]	Mild AD = 27 ± 2			
Huang et al., 2017	CG = 31 (76.5 ± 5.9) [45.2%F]	CG = 27.0 ± 1.2	NINCDS- ADRDA	WCST-64: classical WCST rules, but in a short version. (Heaton, 1981; Kongs et al., 2000).	Achieved Categories: AD = CG
	Mild AD = 31 (78.9 ± 6.3) [64.5%F]	Mild AD = 21.2 ± 3.2	DSM-IV-TR CDR		Perseverative Errors: AD = CG
Redondo et al., 2016	CG = 23 (70.92 ± 4.25) [11M;12F]	CG = 28 ± 1.61	NINCDS-ADRDA	WCST (original version: 128 cards) (Milner, 1963).	% perseverative responses: AD>CG
	DiabetesG = 20 (70.82 ± 3.55) [12M;8F]	DiabetesG = 26.57 ±1.95			% perseverative errors: AD>CG
	AD = 22 (77.74 ± 3.90) [16M;6F]	AD = 23.71 ± 4.25			

*MMSE, Mini-Mental State Examination; AD, Alzheimer Disease Group; WCST, Wisconsin Card Sorting Test; CG, Control Group; DEP, Depression Group; CDR, Clinical Dementia Rating Scale; mWCST, Modified version of the Wisconsin Card Sorting Test; CAMBEX, Cambridge Diagnostic Examination of the Elderly; NINCDS-ADRDA, National Institute of Neurological and Communicative Disorders and Stroke and the Alzheimer's Disease and Related Disorders Association; HD, Huntington's Disease; DRS, Dementia Rating Scale; PDN, Parkinson Disease without Dementia; PDD, Parkinson Disease with Dementia; MCI, Mild Cognitive Impairment; VaD, Vascular Dementia; FTD, Frontotemporal Dementia; KWCST, Keio Version of the Wisconsin Card Sorting Test; aMCI, Amnestic Mild Cognitive Impairment; NIA-AA, National Institute on Aging and Alzheimer's Association*.

Almost all the studies used the diagnostic criteria NINCDS-ADRDA (McKhann et al., 1984) for the probable diagnosis of the AD. Hart et al. (1988) used non-specific criteria and the DRS score to define the AD; Bhutani et al. (1992) took into consideration the criteria of the Cambridge Diagnostic Examination of the Elderly (CAMDEX) (Roth et al., 1986); Chiu et al. (2014) used the criteria of the National Institutes of Health and the Alzheimer's Association, published revised guidelines (NIA-AA) (McKhann et al., 2011). Also, in this case, the participants were tested with the MMSE or DRS to verify the level of cognitive decline, except Chen et al. (2009) who took into account the IQ score to classify patients with the AD.

Concerning the characteristics of the groups, most of the studies matched healthy controls with patients with AD disease about age (considering an average age of over 65) and education. Redondo et al. (2016) and Peltsch et al. (2014) paired patients and healthy people only by considering years of education, groups of patients with AD were older than controls. In the Chiu study (2013), the AD group had lower education than healthy adults. Finally, the study by Kugo et al. (2007) considered patients with AD older and with lower levels of education compared to healthy controls.

Some of these studies compared the WCST performance of patients with the AD with that of patients with MCI, as well as with the healthy elderly group (Tei et al., 1997; Kugo et al., 2007; Chen et al., 2009; Chiu et al., 2014; Peltsch et al., 2014).

To evaluate performance in WCST, all studies used at least two of the following scores: number of completed categories, perseverative errors, total errors, and non-perseverative errors.

The results showed that patients with Alzheimer's disease complete fewer categories (Bondi et al., 1993; Paulsen et al., 1995; Paolo et al., 1996; Stokholm et al., 2006; Kugo et al., 2007; Chen et al., 2009; Chiu et al., 2014) and commit more perseverative errors than healthy people (Hart et al., 1988; Bondi et al., 1993; Paulsen et al., 1995; Nagahama et al., 2003; Kugo et al., 2007; Chen et al., 2009; Redondo et al., 2016). Nevertheless, five studies (Bhutani et al., 1992; Tei et al., 1997; Stokholm et al., 2006; Chiu et al., 2014; Peltsch et al., 2014; Huang et al., 2017) do not identify differences between the numbers of perseverative errors committed by patients compared to those of the control group. Some of these studies (Bhutani et al., 1992; Tei et al., 1997; Peltsch et al., 2014; Huang et al., 2017) did not identify any difference between patients with the AD and the control group even when they considered the number of completed categories.

Redondo et al. (2016) identified a higher percentage of both perseverative errors and perseverative responses in AD patients compared to healthy older; while Nagahama et al. (2003) and Bhutani et al. (1992) showed fewer non-perseverative errors in patients with the AD than in healthy patients. This result could indicate a poor set-shifting capacity in patients affected by Alzheimer's disease that is expressed through a general perseveration of the responses.

### Go/No-Go Task

To analyse the motor inhibition in patients with Alzheimer's disease, four studies (see Table 4) used different versions of the Go/No-Go Task. All the studies used the NINCDS-ADRDA diagnostic criteria for diagnosis (McKhann et al., 1984). All participants (mean age over 65 years in both groups) underwent an assessment of cognitive decline through MMSE (Amieva et al., 2002; Stawarczyk et al., 2012; Rochat et al., 2013) or DRS, (Collette et al., 2007) which confirmed the presence of a higher decline in patients with AD compared to healthy controls. Further, all studies (Amieva et al., 2002; Collette et al., 2007; Stawarczyk et al., 2012; Rochat et al., 2013) used a group of healthy elderly people matched by age and education to the patient group with the AD.

**Table 4:** Some characteristics of the studies that have used the Go/No-Go Task to assess executive dysfunction in patients with Alzheimer's disease.

**Studies**	**Participant N (mean age ±*SD*)**	**MMSE**	**Diagnostic criteria for AD**	**Go/No-Go task typologies**	**Differences between AD group and CG group**		
					**Accuracy (No-Go)**	**Reaction time (Go)**	**Other indices**
Amieva et al., 2002	CG = 28 (75.2 ± 6.6)	CG = 27.6 ± 1.8	NINCDS-ADRDA	*Go Trial (*red circle), *No-Go Trial* (blue triangle). Two errors: (1) wrongly remove the hand from starting point; (2) replace the hand or continue the action touching the No-Go stimulus. Four Data: RT latencies (Go Trial), Reaching time, I error score; II error score.	AD = CG	AD>CG	N/D^[1]^
	Mild AD = 28 (75.8 ± 6.1)	Mild AD = 24.6 ± 1.9					
Collette et al., 2007	CG = 28 (70.6 ± 6.8) [13M;15F]	DRS	NINCDS-ADRDA	*Go Trial* (red circle), *No-Go Trial* (blue triangle). Two errors: 1) wrongly remove the hand from starting point; (2) replace the hand or continue the action touching the No-Go stimulus. Four Data: RT latencies (Go Trial), Reaching time, I error score; II error score.	AD = CG	AD>CG	N/D
	AD = 25 (72.5 ± 5.8) [18M;17F]						
	FTD = 13 (65.7 ± 7.5) [5M;8F]						
Stawarczyk et al., 2012	Study2	MMSE > 21	NINCDS-ADRDA	Two conditions:	AD = CG	AD = CG	N/D
	CG = 16 (76.6 ± 10.6) [7M;9F]			*Simple RT task* (to a stimulus);			
	MildAD = 16 (75.3 ± 10.3) [7M;9F]			2) *Go/No-Go task* (responding as rapid as possible to the previous stimuli but not response to other). Two Data: RT of Go Trials; Accuracy of No-Go Trials (Zimmerman and Fimm, 1994).			
Rochat et al., 2013	CG = 30 (72.05 ± 7.5)	CG = 27.87 ± 1.61	NINCDS-ADRDA	SART: response to a rare target (a number). Three data: (1) number of false alarms (measuring of inhibition); (2) RT of correct responses (measuring processing speed); (3) Coefficient of Variation (measuring attention) (Gay et al., 2008).	AD < CG	AD = CG	CoV: AD>CG
	AD = 30 (72.03 ± 5.9)	AD = 23.27 ± 3.24					

*NIA-AA, National Institute on Aging and Alzheimer's Association*.

The accuracy analysis was evaluated in all studies by considering the number of errors in the No-Go trials (false alarms). Responding to the No-Go stimulus, in fact, is viewed as an error due to impulsivity. Concerning this result, Rochat et al. (2013) shows a higher number of false alarms in the AD group compared to healthy older adults. The other studies (Amieva et al., 2002; Collette et al., 2007; Stawarczyk et al., 2012) did not reveal any difference between patients and controls in the motor inhibition.

Reaction times analysis, in the Go trials, was used to evaluate the global processing speed (Amieva et al., 2002; Collette et al., 2007; Stawarczyk et al., 2012; Rochat et al., 2013). In particular, Collette et al. (2007) and Amieva et al. (2002) confirm a slower performance of patients with AD compared to controls, while Stawarczyk et al. (2012) and Rochat et al. (2013) do not report significant differences between the groups.

The study by Stawarczyk et al. (2012) also analyzed the preservation of inhibitory control through the analysis of reaction times. However, they do not show any difference between controls and patients with Alzheimer's disease. Moreover, Rochat et al. (2013) also considered the Counting coefficient (Standard Deviation/Mean reaction times of the Go Trial) and observed a higher score in patients with AD compared to healthy people, indicating a worse performance to the task.

Overall, the studies that used the Go/No-Go task to assess the motor inhibitory control of patients with Alzheimer's dementia would seem to show a specific heterogeneity in the results. Two of the four studies analyzed (Amieva et al., 2002; Collette et al., 2007) tend to show a general slowdown in response times, which would not indicate a specific deficit in motor inhibition in this task. In contrast, only the study by Rochat and colleagues (Rochat et al., 2013) indicates the presence of an evident executive-motor deficit linked to inhibitory control in patients with Alzheimer's disease.

### Flanker Task

Four studies (Collette et al., 2009; Stawarczyk et al., 2012; Wang et al., 2013; Chen et al., 2017) used different types of flanker tasks inspired by the classic paradigm of Eriksen and Eriksen (Eriksen and Eriksen, 1974), to analyses cognitive inhibition and conflict control in patients with Alzheimer's disease (Table 2).

All the studies used for the probable diagnosis of the AD the diagnostic criteria NINCDS-ADRDA (McKhann et al., 1984).

In all studies, both groups (healthy elderly and elderly with Alzheimer's disease) have an average age over 65 years. For the assessment of the level of cognitive impairment, the scores at MMSE and those of the DRS were taken into consideration. Although the authors do not report in detail the scores obtained by the different groups, they still attest to higher cognitive impairment in patients with the AD. Two of the studies (Wang et al., 2013; Chen et al., 2017), compared patients with the AD with both a healthy control group and a group of subjects classified as MCI.

All the authors recorded reaction times and accuracy of responses in the Flanker Task. Chen et al. (2017) compared the performance between groups only through reaction times; this methodological choice is justified by the fact that the authors consider reaction times as the significant markers of cognitive functioning because it is more closely associated with neural functioning.

The analysis of the reaction times highlighted inconsistent results. Wang et al. (2013) and Chen et al. (2017) reported slower reaction times in patients with Alzheimer compared to healthy elderly, while Collette et al. (2009) and Stawarczyk et al. (2012) did not find significant differences between the two groups.

Analyzing accuracy, only the study by Wang et al. (2013) showed a higher percentage of errors in patients with the AD than in the control group. However, both general reaction times and accuracy give a measure of selective attention, and they do not inform about executive function.

Some of the authors (Collette et al., 2009; Wang et al., 2013) also evaluated the flanker effect index, given by the difference between incongruent and congruent trials in reaction times or accuracy. Specifically, Collette et al. (2009), considering accuracy, reported a higher flanker effect in the patients with the AD than in the control group; Wang et al. (2013) has instead recorded a higher flanker effect in patients the AD compared to the healthy people by considering both reaction times and accuracy. Moreover, Collette et al. (2009) also evaluated the facilitator effect (comparing the accuracy of the congruent trials of the same word and same category conditions to the neutral response condition of Word Flanker Task; see Table 5), this effect was higher in patients with the AD than in the two control groups (young adults and elderly healthy subjects).

**Table 5:** Some characteristics of the studies that have used the Flanker Task to assess executive dysfunction in patients with Alzheimer's disease.

**Studies**	**Participant N (mean age ±*SD*)**	**MMSE**	**Diagnostic criteria for AD**	**Flanker task typologies**	**Differences between AD group and CG group**		
					**Accuracy**	**Reaction time**	**Other indices**
Collette et al., 2009	CG Young = 30 (22.4) [14M;16F]	DRS	NINCDS-ADRDA	Flankers and Target are words. Four different conditions: (1) SW: flankers and target are the same word; (2) NR: flankers are neutral word; (3) SC: flankers and target are of the same category; (4) DR: flankers are the opposite category of the task (incongruent condition)^*^.	AD = CG	AD = CG	Interference effect: ACC: AD>CG
	CG Older = 20 (72.3 ± 5.1) [5M;15F]	CG = 139.1 ± 3.3					Facilitator effect: ACC: AD>CG
	AD = 20 (74 ± 5.8) [6M;14F]	AD = 119.6 ± 8.9					
Stawarczyk et al., 2012	Study2	MMSE > 21	NINCDS-ADRDA	There are a central target (letters) and four flankers (asterisks or letters). Three conditions: (1) facilitator: flankers and target are associated to the same response key; (2) neutral: flanker are asterisks; (3) interferent: target and flankers are associated to different response key.	AD = CG	AD = CG	N/D
	CG = 16 (76.6 ± 10.6) [7M;9F]						
	MildAD = 16 (75.3 ± 10.3) [7M;9F]						
Wang et al., 2013	CG = 16 (69.3 ± 1.8) [9M;7F]	CG = 29.3 ±0.5^*^	NINCDS-ADRDA	Target is an arrow, Flankers are of two types (dots or arrows). Four conditions: (1) target alone, (2) congruent (flankers and target are both arrows and go to the same direction); (3) neutral (flankers are dots); (4) incongruent (flankers and target are both arrows and go to the opposite direction).	AD < CG	AD>CG	Interference effects:
	MCI = 15 (72.9 ± 1.9) [9M;6F]	MCI = 27 ± 0.5^*^					RT = AD>CG ACC = AD < CG
	AD = 7 (68.6 ± 2.9) [3M;4F]	AD = 21.5 ± 0.8						
Chen et al., 2017	CG = 28 (73.7 ± 5.4) [12M;16F]^*^	CDRMC = 0.5	NINCDS-ADRDA	Two different Flanker Task.	N/D	AD>CG	N/D
	MCI = 33 (74.9 ± 5.6) [10M;23F]^*^	AD = 1		*Simple Flanker Task*: a target (arrow) and two flankers (dots). The request is to identify the direction of the arrow.			
	AD = 26 (79.5 ± 6.1) [10M;16F]^*^			*Flanker Reaction Time Task*: a target and two flankers (arrows), with two conditions: congruent (same direction) or incongruent (opposite direction).			

* *There is a significant differences between groups. MMSE, Mini-Mental State Examination; AD, Alzheimer Disease Group; CG, Control Group; DRS, Dementia Rating Scale; NINCDS-ADRDA, National Institute of Neurological and Communicative Disorders and Stroke and the Alzheimer's Disease and Related Disorders; Association; SW, Same-word condition on the Flanker Task; NR, Neutral-response condition on the Flanker Task; SC, Same-category condition on the Flanker Task; DR, Different-response condition on the Flanker Task; ACC, Accuracy (number of correct response); N/D, Data not available for this variable; MCI, Mild Cognitive Impairment; RT, Reaction Time*.

## Discussion

This systematic review was aimed to verify the sensitivity of four golden standard executive functions tasks in catching dysfunctions in these domains in the Alzheimer's disease. Because executive deficits interfere with the performance of daily life activities, by worsening the quality of life of individuals with the AD (Wecker et al., 2000; Collette et al., 2009), it is essential to take this cognitive dimension into account. It is important to note that the recognition of an impairment in the executive functions in the AD is the result of a route change, in fact until a few years ago it was believed that the executive functions were not affected in the pre-clinical stages of dementia (Broks et al., 1996; Razani et al., 2001). Today it is known that these cognitive functions are already damaged prematurely (Binetti et al., 1996; Bondi et al., 2002; Amieva et al., 2004).

A correct choice of the cognitive tasks to use for the assessment of cognitive impairment is a crucial element to take into account. Those considered in this review are the most used in the study of executive functions (Alvarez and Emory, 2006; Duchek et al., 2009; Diamond, 2013) and are widely utilized for cognitive assessment in patients with AD compared to healthy elderly or other pathologies (Collette et al., 2007; Stawarczyk et al., 2012; Peltsch et al., 2014).

They specifically evaluate the capacities of the motor (Go/No-Go Task) and cognitive inhibition (Stroop Task), the conflict control (Flanker Task), and the cognitive flexibility (WCST), the ability to suppress automatic responses and the ability to “resist” to interference (Stroop Task and Flanker task respectively); all skills affected by cognitive decline that are specially compromise in AD.

The Stroop Task seems to be the paradigm that best discriminates between healthy and pathological aging, and it is the most widely used in the research on the AD (Spieler et al., 1996; Belleville et al., 2008; Duchek et al., 2009; Bélanger et al., 2010; McGuinness et al., 2010; Stawarczyk et al., 2012; Peltsch et al., 2014). A neuropsychological assessment including this test can assess the executive system. It could highlight the individual's ability to move the patient's cognitive set, providing a measure of cognitive inhibition and attentional control, and it gives information about the ability to inhibit an overlearned response (i.e., a dominant response, such as the reading) in favor of an unusual stimulus (Spreen and Strauss, 1998). One aspect to consider about this task is that some authors (Hutchison et al., 2010) believe that memory is entailed in the resolution process involved in this task, the decline of which may compromise the overall performance and hinder the ability to focusing attention on the target.

The results obtained with the other tests were inconsistent, but it should be noted that few studies have used them. Concerning the ability of the Wisconsin Card Sorting Test to catch deficits in the cognitive flexibility of Alzheimer's disease, the results are inconsistent. The most critical aspect related to WCST is the complexity of the task. For this reason, some authors (Hart et al., 1988; Bondi et al., 1993; Paulsen et al., 1995; Stokholm et al., 2006) have introduced modified and simplified forms of this test. Contrary to the standard version, on these modified versions of the WSCT, there are fewer cards, and in some case, the subject was informed about a shift in sorting principles. Moreover, there were not ambiguous cards, and the sorting criteria changed after six correct responses. These aspects make the task easier and allow identifying, with higher sensitivity, the cognitive flexibility deficits in the AD (Bondi et al., 1993; Paulsen et al., 1995; Stokholm et al., 2006; Chen et al., 2009). If we considered the modified versions of the test, WCST seems to discriminate between AD patients and healthy subjects (Hart et al., 1988; Bondi et al., 1993; Paulsen et al., 1995; Nagahama et al., 2003; Stokholm et al., 2006; Chen et al., 2009). However, even using a simplified version of the WCST, some studies did not find clear differences between healthy elderly and patients with the AD. Bhutani et al. (1992) believe that the WCST is characterized by a “floor effect” that would not allow discriminating the normal cognitive decline and deterioration typical of dementia, an aspect also reaffirmed by Huang et al. (2017). Moreover, if we consider the studies that compared the performance of patients with the AD with those of elderly people suffering from other dementia diseases (temporal dementia, vascular dementia, Parkinson's with dementia), there are no differences, suggesting that this task not allow discriminating among different forms of dementia (Paulsen et al., 1995; Paolo et al., 1996; Kugo et al., 2007; Li et al., 2011).

The results about the Flanker Task would seem to indicate that this paradigm is not able to highlight a difference between healthy and pathological elderly, especially when reaction times are considered (Collette et al., 2009; Stawarczyk et al., 2012). The analysis of accuracy, instead, has a higher discriminating ability to indicate the actual deterioration in selective attention in patients with the AD (Collette et al., 2009; Wang et al., 2013; Chen et al., 2017). However, if we consider the only two studies that analyzed the flanker effect, both found an impaired conflict control in patients with AD compared to healthy people. The characteristics of the sample represent a weak point of these studies. Two of the four studies did not perform an exact pairing for age and education between patients with the AD and healthy controls. Moreover, the number of participants was very reduced (AD group: *N* = 26 and Control group: *N* = 28; AD group: *N* = 15 and Control group: *N* = 16) (Wang et al., 2013; Chen et al., 2017). For these reasons, and considering the few studies present, it is not possible to exclude that the Flanker Task is sensitive to catch impairment in selective attention and conflict control in Alzheimer's dementia, especially in light of the results obtained at the Stroop Task, which involves attentional aspects similar to those assessed by the Flanker Task (Baddeley and Hitch, 1994).

Also, the results relative to the Go/No-Go task did not indicate whether a deficit in the control of motor inhibition is present or not in patients with AD compared to healthy elderly. Only one study (Rochat et al., 2013) showed a higher number of false alarms in the AD group compared to healthy older adults, indicating an impaired motor inhibition in the AD. The other studies (Amieva et al., 2002; Collette et al., 2007; Stawarczyk et al., 2012) did not reveal any difference between patients and controls in the motor inhibition, suggesting that there not be in AD an executive deficit of this type. This difference between the Rochat's study and the other studies could be due to a different version of the Go/No-Go Task, that required to respond to a rare target (a number); this fact involves a harder inhibitory control ability, that could explain the difference between AD patients and elderly healthy Control Group. However, the comparison between patients with Alzheimer's dementia and patients with different types of dementia showed no difference (Collette et al., 2007; Kugo et al., 2007), suggesting a pattern of motor inhibition common to the different types of dementia.

## Limits and Conclusions

This review highlighted several limitations in the examination of executive functions in Alzheimer's disease. In particular, many studies have used a numerically insufficient sample (Koss et al., 1984; Bhutani et al., 1992; Belleville et al., 2006; Chen et al., 2009; Li et al., 2009, 2011; Coubard et al., 2011; Stawarczyk et al., 2012; El Haj et al., 2013; Wang et al., 2013) others did not consider the level of education of the participants (Spieler et al., 1996; Amieva et al., 2002; Belleville et al., 2006) although this is a variable to be taken into account when analyzing executive functions (Contador et al., 2017). A further problem is related to the assessment of cognitive decline. The different studies have used different scales to evaluate the cognitive decline, and in some studies, the average scores obtained by the various groups are not reported (Paolo et al., 1996; Spieler et al., 1996; Collette et al., 2007; Coubard et al., 2011). Furthermore, the severity of the decline within the AD groups varies a lot as regards the scores at the MMSE (see Tables 2–5). This condition does not allow controlling the influence of these variables on the performance of the tasks used. Furthermore, some studies not matching groups by age, gender and education, and they have not always controlled other aspects (such as the severity of the AD or comorbidity diseases) that could influence task performance (Spieler et al., 1996; Amieva et al., 2002; Collette et al., 2007; Duchek et al., 2009; McGuinness et al., 2010). These dimensions indeed reduce the sensitivity of the instruments in the identification of differences between groups, especially in the case of an analysis of functions that are subject to a physiological decline with age, as also shown by some of the studies considered here that compare elderly control groups with younger control groups (Spieler et al., 1996; Bélanger et al., 2010; Hutchison et al., 2010).

Another critical point of the reviewed studies concerns the use of multiple forms of the same experimental paradigm. These miscellanea of test does not allow for an explicit comparison between the various researches. Therefore, it is difficult to arrive at definite conclusions. This aspect is particularly evident in the studies that have used the WCST. In this case, simplified versions of the test have shown a higher ability to identify differences associated with the AD (Paulsen et al., 1995; Nagahama et al., 2003; Chen et al., 2009), probably because they allow participants to overcome the floor effect.

Concerning the Go/No-Go and the Flanker task, the results were weak in light of the few studies found. Therefore, the systematic use of these tests would be useful, to verify their sensitivity to capture deficits in conflict control and motor inhibition in the AD.

However, regardless of these limitations, at the end of this review emerges an effective executive deficit in the inhibitory control (Chen et al., 2013; Peltsch et al., 2014; Sánchez-Benavides et al., 2014; Huang et al., 2017), and partly also in the cognitive flexibility (Paulsen et al., 1995; Stokholm et al., 2006; Chiu et al., 2014) in the AD. These results allow us to suggest a plausible identikit of executive functioning in Alzheimer's disease, characterized by an impairment in inhibition and cognitive flexibility. There are differences in performance in the various tasks, which could reflect, as previously underlying, differences in the levels of deterioration of the various executive functions analyzed during the AD progression.

In the light of the results of this review that showed a more or less marked discriminatory capacity of the examined tasks for the identification of the executive deficits in Alzheimer's disease, it would be advisable to insert these tasks within neuropsychological batteries. This could allow investigating more entirely and articulately the cognitive functioning of patients affected by the AD.

Overall, this review highlighted the importance of a comprehensive neuropsychological evaluation to allow a clear delineation of the aging profile associated with Alzheimer's disease.

An evaluation of this type could be inserted into pathological aging prevention programs, and it could be useful as a form of monitoring of executive functioning in aging. Furthermore, it could allow identifying the presence of even slight deficits, such as a Mild Cognitive Impairment, that could predict a degenerative disease like the AD. MCI and AD have different diagnostic criteria and different levels of cognitive impairment, for these reasons MCI was not considered in this work. However, it could be useful to conduct a systematic review taking into account the executive performance in MCI specifically.

The results of this review would be to decline it in a meta-analysis that could allow to better understanding the profile of executive functions in the Alzheimer's disease. Furthermore, it would be advisable to carry out a comparative analysis of the different experimental paradigms used to investigate the individual executive functions. This comparison could allow to a better understanding of the results obtained in this work, consenting to conclude whether the results are univocal regardless of the task used or if there is an effect of the type of the task that reinforces or weakens the conclusions to which this review has arrived.

## Author Contributions

AG, MaC and MiC: conception of review, wrote the manuscript; IB, FA and FF literature research, wrote the manuscript. All authors contributed to manuscript revision, read and approved the submitted version.

### Conflict of Interest Statement

The authors declare that the research was conducted in the absence of any commercial or financial relationships that could be construed as a potential conflict of interest.
